# Structure of tRNA splicing enzyme Tpt1 illuminates the mechanism of RNA 2′-PO_4_ recognition and ADP-ribosylation

**DOI:** 10.1038/s41467-018-08211-9

**Published:** 2019-01-15

**Authors:** Ankan Banerjee, Annum Munir, Leonora Abdullahu, Masad J. Damha, Yehuda Goldgur, Stewart Shuman

**Affiliations:** 1 Molecular Biology and Structural Biology Programs, Sloan-Kettering Institute, New York, NY 10065 USA; 2 Chemistry Department, McGill University, Montreal, Quebec H3A0B8 Canada

## Abstract

Tpt1 is an essential agent of fungal tRNA splicing that removes the 2′-PO_4_ at the splice junction generated by fungal tRNA ligase. Tpt1 catalyzes a unique two-step reaction whereby the 2′-PO_4_ attacks NAD^+^ to form an RNA-2′-phospho-ADP-ribosyl intermediate that undergoes transesterification to yield 2′-OH RNA and ADP-ribose-1″,2″-cyclic phosphate products. Because Tpt1 is inessential in exemplary bacterial and mammalian taxa, Tpt1 is seen as an attractive antifungal target. Here we report a 1.4 Å crystal structure of Tpt1 in a product-mimetic complex with ADP-ribose-1″-phosphate in the NAD^+^ site and pAp in the RNA site. The structure reveals how Tpt1 recognizes a 2′-PO_4_ RNA splice junction and the mechanism of RNA phospho-ADP-ribosylation. This study also provides evidence that a bacterium has an endogenous phosphorylated substrate with which Tpt1 reacts.

## Abstract (web summary)

Tpt1 catalyzes the final essential step in yeast tRNA splicing and is a potential antifungal target. Here the authors provide structural insights into how Tpt1 recognizes a 2’-PO_4_ RNA splice junction and the mechanism of RNA phospho-ADP-ribosylation.

## Introduction

**Fig. 1:**
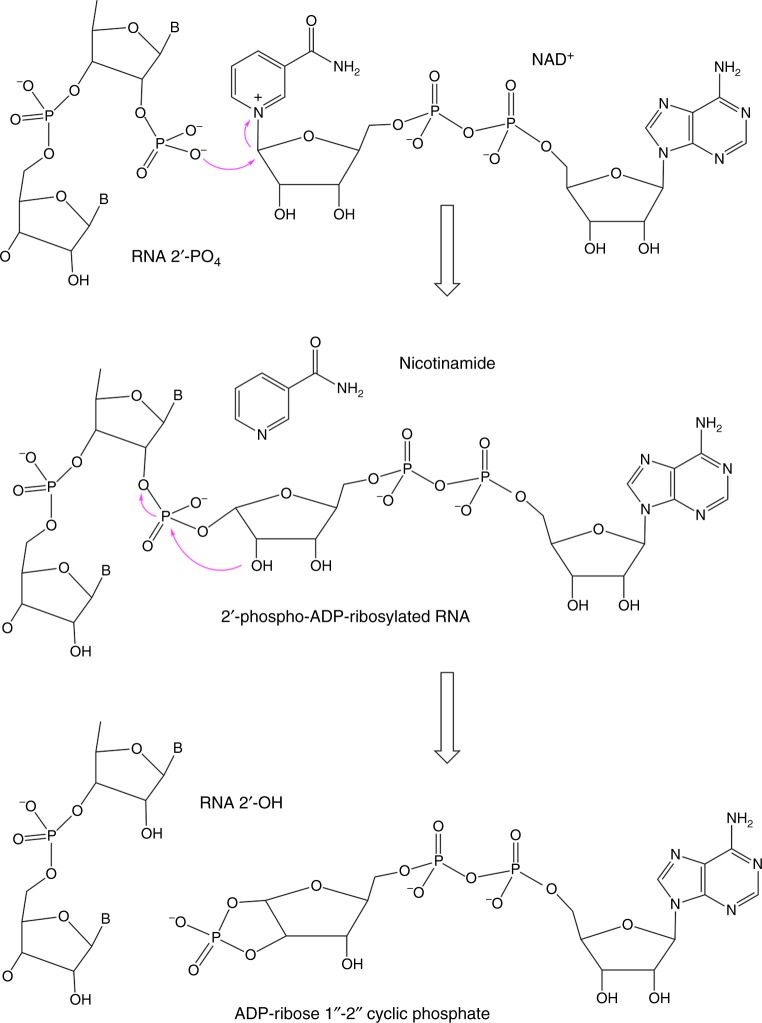
Two-step Tpt1-catalyzed mechanism of 2′-PO_4_ removal from a 2′-PO_4_, 3′-5′ phosphodiester RNA junction. The Tpt1 reaction pathway comprises the two chemical steps shown in which: (1) the RNA 2′-PO_4_ reacts with NAD^+^ to expel nicotinamide and form a 2′-phospho-ADP-ribosylated RNA intermediate; and (2) transesterification of the ADP-ribose 2′′-OH to the RNA 2′-PO_4_ displaces the RNA 2′-OH and generates ADP-ribose-1″,2″-cyclic phosphate

Tpt1 is a fascinating enzyme that transfers an internal RNA 2′-monophosphate (2′-PO_4_) to NAD^+^ to form a 2′-OH RNA and ADP-ribose-1″,2″-cyclic phosphate^1^. The Tpt1 mechanism comprises two chemical steps in which: (i) the RNA 2′-PO_4_ reacts with NAD^+^ to expel nicotinamide and form a 2′-phospho-ADP-ribosylated RNA intermediate; and (ii) transesterification of the ADP-ribose 2′′-OH to the RNA 2′-PO_4_ displaces the RNA 2′-OH and generates ADP-ribose-1″,2″-cyclic phosphate (Fig. 1)^2–5^. Tpt1 was discovered by Eric Phizicky and colleagues as an essential component of the fungal tRNA splicing pathway^6–9^, which characteristically generates a 2′-PO_4_, 3′-5′ phosphodiester splice junction during the tRNA ligation reaction^10,11^. Archaea and metazoa have Tpt1 homologs^12^, notwithstanding that the pathway of archaeal and metazoan tRNA exon ligation is entirely different from that of fungi and does not result in a junction 2′-PO_4_^13^. Tpt1 homologs are also widely prevalent in bacterial species, many of which have no known intron-containing tRNAs and/or no known pathways to generate RNAs with internal 2′-PO_4_ modifications. Bacterial and human Tpt1 homologs have NAD^+^-dependent RNA 2′-phosphotransferase activity in vitro and can genetically complement an otherwise lethal *tpt1*∆ deletion in *Saccharomyces cerevisiae*, signifying that they are capable of removing the tRNA splice junction 2′-PO_4_ in vivo^2,5,12,14^.

Whereas it is not obvious what reactions Tpt1 performs in taxa that lack a fungal-type RNA ligase, it is clear that Tpt1 is not essential for their viability and fitness under laboratory conditions, insofar as genetic ablation of *E. coli* Tpt1 (also known as KptA) and mouse Tpt1 has no phenoptypic consequences^12,15^. These findings underscore the high value of Tpt1 as a target for antifungal drug discovery, predicated on pharmacological inhibition of Tpt1′s essential function in fungal tRNA splicing and its contribution to the the fungal unfolded protein response^8,9,16,17^.

The lack of a structural snapshot of Tpt1 at relevant steps along the reaction pathway is a major knowledge gap in fungal tRNA metabolism and an impediment to inhibitor discovery and design. Here we close this gap by attaining a 1.4 Å crystal structure of Tpt1, providentially captured in a complex with ADP-ribose-1″-phosphate in the NAD^+^ site (generated during protein production in *E. coli*) and the pAp moiety of coenzyme A in the RNA site. This structure, which mimics the step 2 product complex of the Tpt1 reaction, yields keen insights into how Tpt1 recognizes a 2′-PO_4_ RNA splice junction. It also provides evidence that a bacterium has a phosphorylated substrate with which Tpt1 reacts.

## Results

### Crystallization of Tpt1 and structure determination

**Fig. 2:**
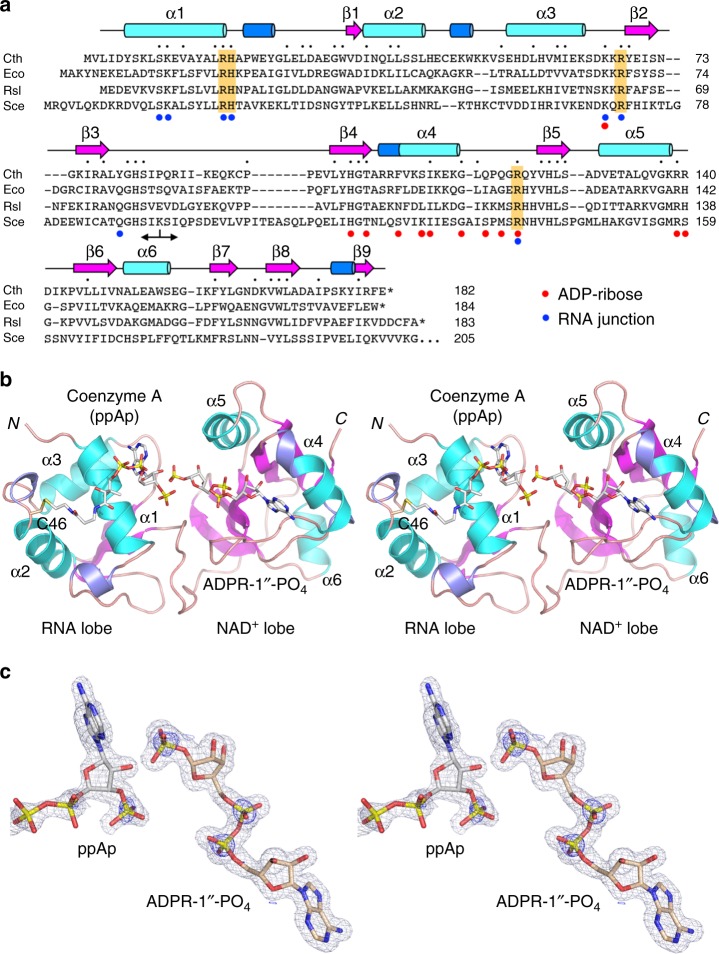
Overview of the Tpt1 structure and active site ligands. **a** The primary structure of CthTpt1 is aligned to those of Tpt1 enzymes from *Escherichia coli* (Eco), *Runella slithyformis* (Rsl), and *Saccharomyces cerevisiae* (Sce). Positions of amino acid side chain identity or similarity in all four proteins are indicated by black dots above the CthTpt1 sequence. Gaps in the alignment are indicated by dashes. The constituents of a conserved Arg-His-Arg-Arg tetrad essential for Tpt1 activity are highlighted in gold shading. The secondary structure elements of CthTpt1—magenta β strands, cyan α helices, and blue 3_10_ helices—are displayed above the CthTpt1 sequence. The boundary between the N-terminal RNA lobe (aa 1–85) and the C-terminal NAD^+^ lobe (aa 86–182) is demarcated by a two-headed arrow below the alignment. Amino acids that contact the ADP-ribose moiety in the NAD^+^ site are denoted by red dots below the alignment. Amino acids that contact the RNA splice junction (the pAp and the 1″-PO_4_) are denoted by blue dots. **b** Stereo view of the CthTpt1 tertiary structure, depicted as a cartoon model with magenta β strands, cyan α helices, and blue 3_10_ helices. The *N* and *C* termini are indicated and the α helices are numbered. The ADP-ribose-1″-PO_4_ and coenzyme A ligands are rendered as a stick models with gray carbons. **c** A stereo view of the simulated annealing omit 2Fo-Fc map of the ADP-ribose-1″-PO_4_ and pAp ligands in the active site, contoured at 1σ, is shown in gray mesh. Anomalous difference density for the phosphorus atoms of ADP-ribose-1″-PO_4_ and pAp, contoured at 3σ, is shown in blue mesh. The simulated annealing omit 2Fo-Fc and anomalous difference density maps were calculated with both ligands omitted from the model. Anomalous peaks for the sulfur atoms of Met60 and Cys94 were apparent at 4σ but are not shown because they are remote from the two active site ligands

We succeeded in growing crystals of Tpt1 from the mesothermophilic bacterium *Clostridium thermocellum* (Cth) using recombinant CthTpt1 produced in *E. coli*. (see Supplementary Fig. 1A). The primary structure of the 182-aa CthTpt1 polypeptide is homologous to that of the Tpt1 enzymes from *E. coli*, *Runella slithyformis*, and *S. cerevisiae*, which have been well-characterized biochemically and genetically^2–5,12,14^. As shown in Fig. 2a, these four Tpt1s have 50 positions of amino acid side chain identity/similarity, including a constellation of four conserved amino acids (an Arg-His-Arg-Arg tetrad) essential for Tpt1 activity in vivo and in vitro^5,14^. Prior to crystallization, the biochemical activity of CthTpt1 was verified by its reaction with a synthetic 5′ ^32^P-labeled 6-mer RNA with an internal 2′-PO_4_ group, resulting in its quantitative conversion to a 2′-OH product (see Supplementary Fig. 1B). Removal of the 2′-PO_4_ from the 6-mer RNA required added NAD^+^, but not a divalent cation. Hexagonal CthTpt1 crystals were in space group P6_1_22 and diffracted X-rays to 1.4 Å resolution. The refined Tpt1 structure (*R*_work_/*R*_free_ 16.16/18.57) comprised a continuous polypeptide from Leu3 to Glu182, consisting of nine β strands, six α helices, and four 3_10_ helices (Fig. 2a, b), organized into two distinct lobes spanning amino acids 1–85 and 86–182, respectively (Fig. 2b).

### Surprise active site ligands mimic a step 2 product complex

The 1.4 Å electron density map revealed two non-protein ligands bound to Tpt1 that were acquired during production of the recombinant protein in *E. coli*. The molecule associated with the C-terminal lobe was unambiguously modeled as ADP-ribose-1″-phosphate based on the electron density and the anomalous difference peaks overlying the three phosphorus atoms (Fig. 2c). The parsimonious explanation for the presence of this molecule is that recombinant Tpt1 catalyzed a reaction of NAD^+^, present in the *E. coli* cytosol at a concentration of 2.6 mM^18^, with an endogenous *E. coli* phosphorylated substrate (conceivably a 2′-PO_4_ branched RNA) to form ADP-ribose-1″,2″-cyclic phosphate, which was in turn hydrolyzed in situ to ADP-ribose-1″-phosphate during the long time-frame of Tpt1 purification and crystal growth. Henceforth, we refer to the C-terminal module of Tpt1 as the NAD^+^ lobe.

The molecule associated with the N-terminal lobe was convincingly modeled as coenzyme A (CoA) based on: (i) the density of the terminal ppAp moiety in the Tpt1 active site and the anomalous difference peaks overlying the phosphorus atoms of the terminal pAp moiety (Fig. 2c), (ii) the electron density flanking ppAp corresponding to the pantetheine moiety of CoA (see Supplementary Fig. 2), and (iii) covalent attachment of the terminal CoA sulfur to Tpt1 via a disulfide bond to Cys46 (Fig. 2b and Supplementary Fig. 2). The pAp terminus of CoA is a mimetic of the dephosphorylated RNA product of the canonical Tpt1 reaction pathway. Therefore, we will refer to the N-terminal module as the RNA lobe. The 4′-phosphopantetheine moiety of CoA drapes over the surface of the RNA lobe between its point of covalent attachment to Cys46 and occupancy of the RNA site by the pAp nucleotide of CoA (Fig. 2b). Direct contact between the 4′-phosphopantetheine and Tpt1 is limited to a single-hydrogen bond from the CoA N4 atom to Glu12 Oε2.

We envision that: (i) an endogenous substrate dissociated from Tpt1 after transfer of its phosphate to NAD^+^ to form ADP-ribose-1″,2″-cyclic phosphate; (ii) the RNA product site was then fortuitously occupied by the pAp moiety of CoA, present in the *E. coli* cytosol at 1.3 mM concentration^18^; and (iii) CoA remained bound to Tpt1 during purification because it was covalently tethered to Cys46. We do not suppose that these two ligands are associated with all of the CthTpt1 protein molecules in the recombinant enzyme preparation (which is active as an RNA 2′-phosphotransferase), but rather that the ligand-bound sub-population of CthTpt1 is the unique form that crystallized.

The Cys46 residue of CthTpt1, to which CoA is attached, is conspicuously not conserved in other Tpt1 homologs (Fig. 2a), thereby underscoring the serendipity of covalent capture of CoA bound to CthTpt1. To explore this point, we mutated Cys46 to serine, produced Tpt1-C46S in *E. coli*, crystallized the C46S mutant, and determined its structure at 1.6 Å resolution (Supplementary Table 1). The Tpt1-C46S structure was notable for the following: (i) its tertiary structure was virtually identical to that of wild-type Tpt1; (ii) ADP-ribose-1″-phosphate was bound to the NAD^+^ lobe; and (iii) CoA was absent from the RNA lobe.

### NAD^+^ recognition

**Fig. 3:**
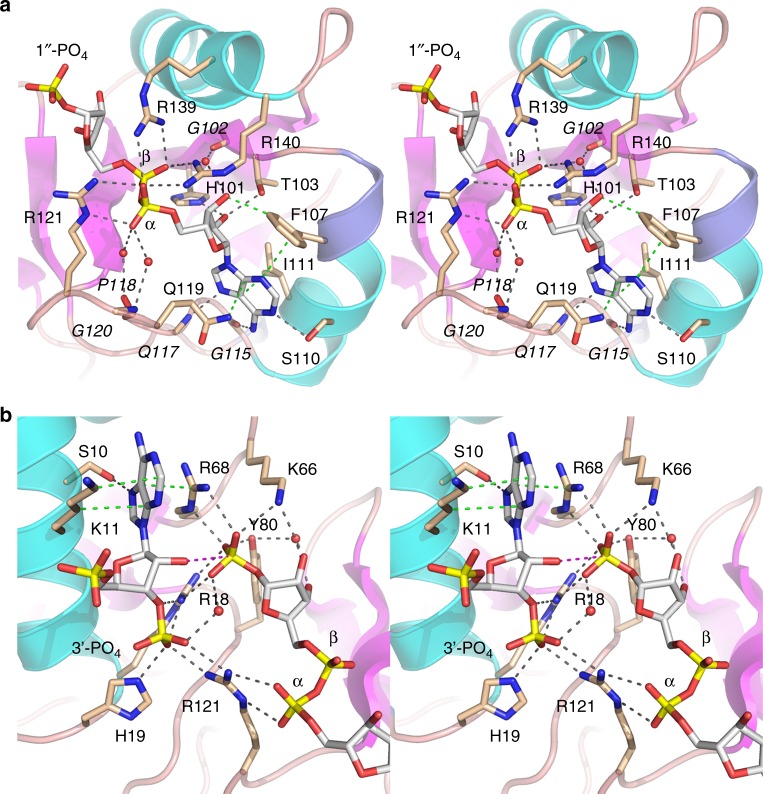
Tpt1 active site. Stereo views of the atomic interactions of Tpt1 with the ADP-ribose moiety of the ADP-ribose-1″-PO_4_ product **a** and the pAp and 1″-PO_4_ moieties **b**. Selected Tpt1 amino acids are depicted as stick models with beige carbons. The ligands are depicted as stick models with gray carbons. Waters are depicted as red spheres. Hydrogen bonds are denoted by black dashed lines and van der Waals contacts by green dashed lines. The distance (3.5 Å) from the ribose 2′-O to the 1″-P in **b** is indicated by a magenta dashed line. Amino acids that make side-chain contacts to the ligand are labeled in plain font; those that make main-chain contacts to the ligands are labeled in italics

The atomic interactions of Tpt1 with the ADP-ribose moiety of the ADP-ribose-1″-phosphate product (Fig. 3a) illustrate the basis for NAD^+^ substrate recognition. The adenosine nucleoside is in an *anti* conformation with the purine nucleobase sandwiched between Gln119 and Ile111. Adenine specificity is conferred by three hydrogen bonds to the purine ring nitrogens: to adenine-N1 from Ser110; from adenine-N6 to the Gly115 carbonyl; and from the Gln117 main-chain amide to adenine-N7 (Fig. 3a). The adenosine ribose 2′-OH makes bifurcated hydrogen bonds with His101-Nε and Thr103-Oγ. Phe107 makes van der Waals contact with the adenosine ribose O3′ and adenine-N3 (Fig. 3a). The adenosine ribose 3′-OH is bridged to the β phosphate via a water that is in turn engaged by the Gly102 carbonyl. The α and β phosphates are held in a cage of bidentate hydrogen bonds from the guanidium nitrogens of Arg121, Arg139, and Arg140 to the non-bridging phosphate oxygens (Fig. 3a). Also, the Pro118 carbonyl and the Gly120 amide make water-mediated contacts to the α phosphate.

### RNA and 2′-PO_4_ recognition

**Fig. 4:**
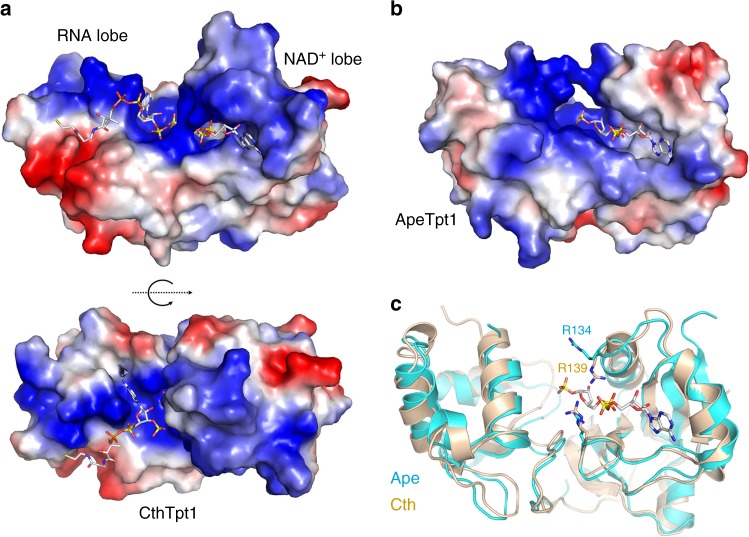
Surface electrostatics and comparison to ApeTpt1 apoenzyme. **a** Surface electrostatic model of the CthTpt1 protein generated in Pymol. ADP-ribose-1″-PO_4_ and CoA ligands are depicted as stick models. The top view highlights positive potential (blue) across the RNA and NAD^+^ lobes surrounding the ligands, with ADP-ribose-1″-PO_4_ being encased below an “arch″ formed by two arginines. The bottom view shows the adenine of pAp wedged into a groove on the positive surface. **b** Surface electrostatic model is shown of the *Aeropyrum pernix* (Ape) Tpt1 apoenzyme (pdb 1WFX) with ADP-ribose-1″-PO_4_ imported from the CthTpt1 structure. **c** Superposition of the CthTpt1 (beige) and ApeTpt1 (cyan) structures. The arginines that form the arch over ADP-ribose-1″-PO_4_ in CthTpt1 are shown as stick models. Side chain movement of ApeTpt1 Arg 134 versus CthTpt1 Arg139 gives access to the NAD^+^ binding pocket

The interactions of Tpt1 with the pAp moiety of CoA and the 1″-phosphate of ADP-ribose-1″-phosphate (Fig. 3b) reveal the basis for splice junction recognition and step 2 catalysis. The 1″-PO_4_ of ADP-ribose-1″-phosphate is the α anomer configuration of the ribose C1″ (Fig. 3b), reflecting the expected stereochemical inversion of the β-NAD^+^ substrate during the first step of the Tpt1 reaction pathway (Fig. 1). The 1″-PO_4_ is situated adjacent to the ribose 2′-OH of pAp (O2′–P distance of 3.5 Å), consistent with the 1″-PO_4_ corresponding to the 2′-PO_4_ of an RNA splice junction after its transfer to ADP-ribose in the second step of the Tpt1 pathway (Fig. 1). The transferred phosphate is encased within a hydrogen bond network to its phosphate oxygens, entailing single-hydrogen bonds from Tyr80 to Lys66 and bidentate hydrogen bonds from Arg18 to Arg68 (Fig. 3b). The vicinal 3′-PO_4_ at the splice junction receives hydrogen bonds to its oxygens from His19, Arg18, and Arg121 (Fig. 3b). The nucleobase at the splice junction fits into a groove on the enzyme surface (Fig. 4a lower panel) where it is sandwiched between a π-cation stack with Arg68 and the aliphatic segment of the Lys11 side chain (Fig. 3b). Ser10-Oγ donates a hydrogen bond to the purine N7 position. Lys11-Nζ is positioned 3.5 Å away from a 5′-PO_4_ oxygen of pAp (Fig. 3b).

Note that the Arg18, His19, Arg68, and Arg121 side chains that engage the junction 3′-PO_4_ and 2′-PO_4_ are the CthTpt1 equivalents of the invariant Arg-His-Arg-Arg tetrad that is essential for the activity of other bacterial and fungal Tpt1 enzymes^5,14^. Our structure makes clear that this tetrad is a key part of the Tpt1 active site and highlights the junction 3′-PO_4_ and 2′-PO_4_ as the crucial RNA substrate recognition elements. Furthermore, the structure suggests that Arg18 and Arg66 promote step 2 catalysis via stabilization of the transition state of the transferred 2′-PO_4_. This mechanism is consistent with the finding that alanine mutations of Arg16 and Arg64 in RslTpt1 (equivalent to Arg18 and Arg66 in CthTpt1) slow the rate of single-turnover step 2 catalysis by 710-fold and 210-fold, respectively, *vis-à-vis* the wild-type enzyme^5^. By contrast, alanine mutation of RslTpt1 His17 (equivalent to CthTpt1 His19 that contacts only the junction 3′-PO_4_) elicits a modest threefold decrement in the single-turnover step 2 rate constant^5^.

### Surface electrostatics suggest an RNA binding surface

A surface electrostatic model of the Tpt1 protein (Fig. 4a) highlights a broad swath of positive potential across the RNA and NAD^+^ lobes surrounding the pAp and ADP-ribose-1″-phosphate ligands. Within the RNA lobe, the adenine of pAp, exemplifying the splice junction base, wedges into a positive pocket (Fig. 4a, bottom panel) lined by Lys11 and Arg68. We infer that the junction base is flipped out of the RNA substrate during engagement of the junction phosphates in the Tpt1 active site. The 3′-PO_4_ of pAp is pointing toward the positive surface of the NAD^+^ lobe above the buried ADP-ribose-1″-phosphate, suggesting a plausible path for an RNA segment distal to the splice junction.

### An imputed conformational change upon NAD^+^ binding

The ADP-ribose-1″-phosphate is topologically encased in the CthTpt1 NAD^+^ lobe by virtue of a bridge formed by Arg121 and Arg139 over the ribose-5″-diphosphate moiety (Fig. 4a, top panel). This closed conformation in the product complex implies that the enzyme needs to “open up″ to allow ingress of the NAD^+^ substrate (and egress of the ADP-ribose-1″-2″-cyclic phosphate product). The nature of this conformational step is hinted at by comparison of the ligand-bound CthTpt1 structure to that of the ligand-free apoenzyme form of *Aeropyrum pernix* (Ape) Tpt1 (pdb 1WFX [https://www.rcsb.org/structure/1wfx])^19^. A comparison in DALI^20^ of the CthTpt1 and ApeTpt1 protein structures yielded a Z score of 22.1 with a rmsd of 1.9 Å at 173 Cα positions with 34% identity. Superposition of the ADP-ribose-1″-phosphate from CthTpt1 onto a surface model of the ApeTpt1 apoenzyme revealed that the NAD^+^-binding site is fully surface exposed (Fig. 4c). An alignment of the CthTpt1 and ApeTpt1 tertiary structures pointed to side-chain movement of an NAD^+^-binding arginine (Arg139 in CthTpt1, corresponding to Arg134 in ApeTpt1) as a likely key to opening the NAD^+^ entry path (Fig. 4c).

### Homology of the Tpt1 NAD^+^ lobe to ADP-ribosylating toxins

**Fig. 5:**
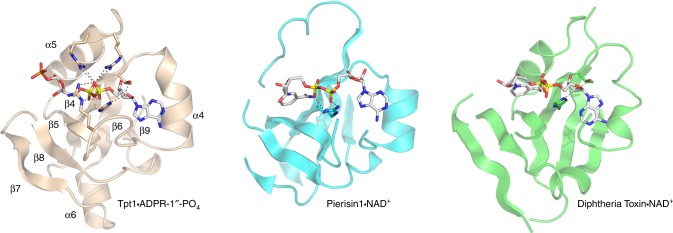
Homology of the Tpt1 NAD^+^ lobe to ADP-ribosylating toxins. A side-by-side alignment of the homologous segments of the Tpt1•ADPR-1″-PO_4_ (beige), Pierisin1•NAD^+^ (pdb 5H6J, cyan), and diphtheria toxin•NAD^+^ (pdb 1TOX, green) structures is shown. The ligands are depicted as stick models with gray carbons. Amino acid contacts to the ADP-ribose moieties are indicated by dashed lines

The fold of the Tpt1 NAD^+^ lobe with ADP-ribose-1″-phosphate bound (depicted in Fig. 5) consists of a six-strand antiparallel β-sheet (with topology β7↑•β8↓•β5↑•β4↓•β6↑•β9↓) and three α helices (α4, α5, α6). A DALI search of the Protein Database with the CthTpt1 structure recovered multiple “hits” to the C-terminal NAD^+^ lobe, all of which belonged to the family of mono-ADP-ribosylating toxins. In each case, the homology was limited to the NAD^+^-binding modules of the respective proteins. The top hit was to *Mycoplasma pneumoniae* CARDS toxin crystallized without NAD^+^ bound (pdb 4TLV; Z score 7.7; rmsd of 1.9 Å at 73 Cα positions with 21% identity)^21^. Structures of Tpt1-homolgous toxins with NAD^+^ bound include: the DNA-targeting toxin Pierisin1 from the cabbage butterfly *Pieris rapae* (pdb 5H6J; Z score 5.2; rmsd of 3.7 Å at 88 Cα positions with 14% identity)^22^ and diphtheria toxin (pdb 1TOX and pdb 1MDT; Z score 4.7; rmsd of 4.5 Å at 83 Cα positions with 16% identity)^23^. A side-by-side alignment of the homologous segments of the Tpt1•ADPR-1″-PO_4_, Pierisin1•NAD^+^, and diphtheria toxin•NAD^+^ structures is shown in Fig. 5. The shared fold embraces the six-strand β-sheet and equivalent of the Tpt1 α4 helix. The ADP-ribose components of the ligand are similarly draped across the β-sheet with the adenosine nucleoside on the right. However, the conformation of the adenosine nucleoside and the protein contacts to ADP-ribose vary significantly. Whereas adenosine is in the *anti* conformation in Tpt1 and Pierisin1, it is in the *syn* conformation in diphtheria toxin (Fig. 5). Tpt1 and diphtheria toxin engage the adenosine 2′-OH via a pair of hydrogen bonds from the His and Thr side chains of a conserved HGT motif (^101^HGT^103^ in Tpt1; ^21^HGT^23^ in diphtheria toxin) (Fig. 5). By contrast, there are no atomic contacts to the adenosine 2′-OH in the Pierisin1•NAD^+^ structure. Rather, in Pierisin1, an arginine (Arg70) occupies the position of His101 in Tpt1 and this Arg70 side chain makes a bidentate interaction with the NAD^+^ α and β phosphates (Fig. 5). There are no atomic contacts to the NAD^+^ phosphates in the diphtheria toxin•NAD^+^ structure. The cage of three arginines around the ADP-ribose α and β phosphates is apparently a distinctive feature of Tpt1, in which one of the arginines (Arg121) does double duty in engaging the RNA splice junction 3′-PO_4_.

### Tpt1 RNA lobe

The tertiary structure of the Tpt1 RNA lobe (see Supplementary Fig. 3) consists of a three-strand antiparallel β-sheet (with topology β2↑•β3↓•β1↑) packed against a three helix bundle (α1, α2, and α3). A DALI search of the Protein Database with the CthTpt1 RNA lobe recovered multiple “hits″ to protein domains with winged helix folds. The top hit was to the N-terminal winged helix domain of human rheumatic disease autoantigen La (pdb 1YTY; *Z*-score 7.1; rmsd of 3.8 Å at 72 Cα positions with 21% identity), an RNA-binding protein that recognizes the UUU_OH_ 3′ termini of RNA polymerase III transcripts^24^. The superimposed Tpt1 and La domains are shown in Supplementary Fig. 3. A key point is that the three essential active site amino acids in the Tpt1 RNA lobe that interact with the pAp and ADP-ribose-1″-phosphate ligands and are conserved among Tpt1 homologs (Arg18, His19, and Arg68) are not conserved in human La. We surmise that Tpt1 evolved its unique specificity and catalytic mechanism *vis-à-vis* other mono-ADP-ribosyltransferases^25^ via fusion of a specialized NAD^+^ lobe to a specialized winged helix domain.

### Structure-guided mutagenesis

**Fig. 6:**
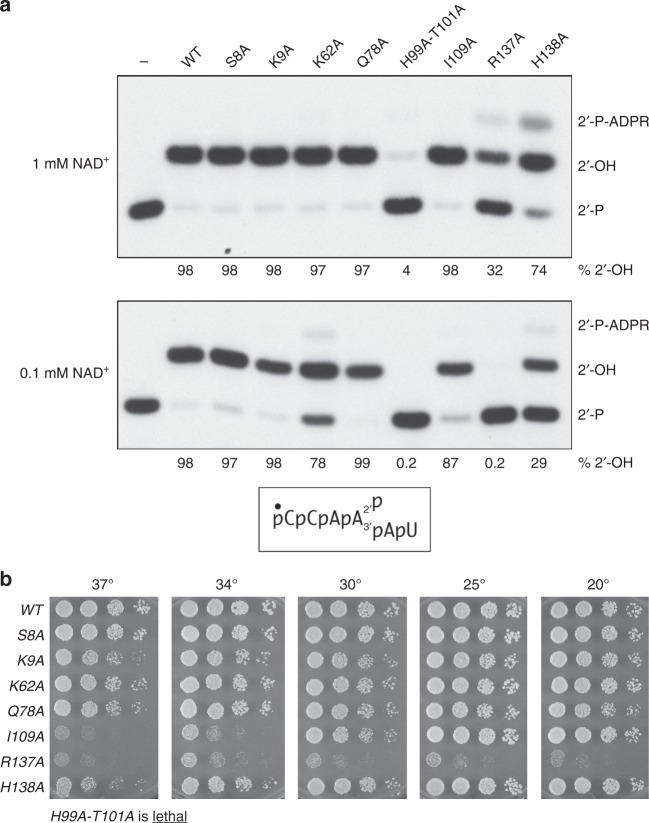
Structure-guided mutagenesis. **a** Reaction mixtures containing 100 mM Tris-HCl (pH 7.5), either 1 mM or 0.1 mM NAD^+^ as specified, 0.2 µM (2 pmol) 5′ ^32^P-labeled 6-mer 2′-PO_4_ RNA (shown at bottom), and 0.01 µM (0.1 pmol) wild-type or mutant RslTpt1 as specified were incubated for 30 min at 37 °C. Tpt1 was omitted from the control reaction mixture in lane –. The reaction products were analyzed urea-PAGE and visualized by autoradiography. The positions of the 6-mer 2′-PO_4_ RNA substrate (2′-P), 2′-P-ADPR RNA intermediate, and 2′-OH RNA product (2′-OH) are indicated on the right. The extents of 2′-OH RNA product formation (expressed as percent of total labeled RNA) are indicated below the lanes. **b** *tpt1*∆ complementation was performed by plasmid shuffle as described under Methods. Serial dilutions of the indicated *S. cerevisiae tpt1*∆ *RslTPT1* strains were spot-tested for growth on YPD agar at the temperatures specified

Having previously biochemically and genetically characterized the role of the active site Arg-His-Arg-Arg tetrad of *Runella slithyformis* (Rsl) Tpt1 via alanine scanning^5^, we elected to continue using RslTpt1 for structure-activity studies, now guided by the CthTpt1 crystal structure. Here we performed an alanine scan of RslTpt1 amino acids Ser8, Lys9, Lys62, Gln78, His99, Thr101, Ile109, Arg137, and His138 (equivalent to Ser10, Lys11, Lys66, Tyr80, His101, Thr103, Ile111, Arg139, and Arg140 in CthTpt1; Fig. 2a). Seven of these residues were mutated singly to alanine. His99 and Thr101 were doubly mutated in light of their potentially functionally redundant hydrogen bonds to the ADP-ribose 2′-OH, suggested by previous mutational analysis of *S. cerevisiae* Tpt1^14^. The Rsl1Tpt1-Ala proteins were purified (see Supplementary Fig. 4) and tested for RNA 2′-phosphotransferase activity in the presence of 1 mM NAD^+^ (Fig. 6a). Under these conditions, the wild-type, S8A, K9A, K62A, Q78A, and I109A proteins effected near-quantitative removal of the 2′-PO_4_ to form a 2′-OH product. By contrast, the H99A-T101A protein was severely defective, converting only 4% of input substrate to 2′-OH product. The H138A and R137A mutants were partially compromised, as evinced by their lower yield of 2′-OH product and accumulation of the 2′-phospho-ADP-ribosylated RNA intermediate (Fig. 6a). When the same experiment was performed at 0.1 mM NAD^+^, we found that there was little or no change in the yield of 2′-OH product by the WT, S8A, K9A, and Q78A proteins. However, the R137A mutant lost all activity when the NAD^+^ concentration was reduced (Fig. 6a). Lower NAD^+^ exacerbated the defect of H138A and exposed K62A as less active *vis-à-vis* wild type. The constitutive and NAD^+^ concentration-sensitive defects accompanying loss of the side chains of His99/Thr101, Arg127, His138, and Lys62 in Rsl1Tpt1 are in accord with the atomic interactions with NAD^+^ made by the corresponding side chains in CthTpt1.

The *RslTPT1-Ala* alleles on *CEN HIS3* plasmids were tested by plasmid shuffle for complementation of a *S. cerevisiae tpt1*∆ p[*CEN URA3 ScTPT1*] strain^5,14^. The *RslTPT1-(H99A-T101A)* mutant was unable to give rise to FOA-resistant colonies and was therefore deemed lethal in vivo. The lack of in vivo function of this mutant is consistent with its severe activity defect in vitro. The *tpt1*∆ *RslTPT1-Ala* strains that passed FOA selection were spot tested for growth on YPD agar at 20, 25, 30, 34, and 37 °C in parallel with the *tpt1*∆ *RslTPT1-WT* control (Fig. 6b). The *R137A* mutant, which was slow to form colonies on FOA during initial selection, was barely viable on YPD agar at any temperature, as gauged by reduced colony number and microscopic colony size. Here again, the in vivo defect of R137A was consonant with its deleterious effect on Tpt1 activity in vitro. The other six *RslTPT1-Ala* strains grew as well as wild type at 25 °C. Whereas *S8A*, *K62A*, and *Q78A* cells grew well at all temperatures, the *I109A* strain displayed a tight temperature-sensitive (*ts*) phenotype, seen as failure to grow at 37 °C and poor growth at 34 °C. The *H138A* and *K9A* mutants had a modest *ts* phenotype, forming smaller colonies than wild type at 37 °C (Fig. 6b).

## Discussion

The present study enhances our understanding of the structural basis for RNA 2′-phosphate removal by Tpt1 enzymes, wherein attack of the junction 2′-phosphate on the nicotinamide ribose of NAD^+^ leads to the formation of a 2′-phospho-ADP-ribosylated RNA intermediate that is subsequently converted to an RNA_2′OH_ product via transesterification of the phosphate to the ribose O2″. We were fortunate in crystallizing CthTpt1 in a ligand-bound state that mimics the product complex after transfer of the junction phosphate to the C1″ atom of ADP-ribose and retention of the 3′-PO_4_, 2′-OH junction nucleotide in the RNA site. We thereby identified the essential Tpt1 functional groups responsible for recognition of the splice junction, via an elaborate network of hydrogen-bonding to the 3′-PO_4_ and 2′-PO_4_ moieties by the conserved and essential Arg-His-Arg-Arg tetrad. Two of the essential tetrad arginines that coordinate the 1″-PO_4_ in the structure (Arg18 and Arg68 in CthTpt1) are implicated (via kinetic effects of the corresponding RslTpt1 R16A and R64A mutants^5^) in stabilizing the negative charge on the phosphorane transition state of the 2′-1″ phosphodiester during the step 2 transesterification reaction of the Tpt1 pathway. Yet, these same two arginines play distinct roles during step 1 of the Tpt1 pathway. The RslTpt1 R64A mutation has little impact on the step 1 rate constant (threefold reduction compared to wild-type Tpt1). By contrast, the RslTpt1 R16A change slows the step 1 rate by 380-fold^5^. The structure rationalizes this disparity, insofar as Arg18/Arg16 engages both the 3′-PO_4_ and 2′-PO_4_ at the RNA splice junction. It thereby facilitates RNA binding and proper orientation of the 2′-PO_4_ for its attack on the C1″ position of NAD^+^ (Fig. 1). A conserved lysine that contacts the 1″-PO_4_ (Lys66 in CthTpt1; Lys62 in RslTpt1; Lys69 in SceTpt1) is not essential for the activity of RslTpt1 (Fig. 6) or SceTpt1^14^, likely because its role is ancillary to that of the two essential arginines that engage the transferred 2′-PO_4_ of the junction.

Here we also gained new insights into the basis for NAD^+^ recognition by Tpt1 enzymes, highlighting the importance of basic amino acids that cage the NAD^+^ phosphates and the conserved HGT motif that engages the NAD^+^ 2′-OH via the histidine and threonine side chains. Whereas the fold of the Tpt1 NAD^+^ lobe is related to the mono-ADP-ribosyltransferase toxins, we find that its basis for NAD^+^ recognition is distinctive and entails more extensive atomic contacts than those seen in the available toxin•NAD^+^ structures.

The structural homology between fungal and bacterial Tpt1 enzymes and their functional orthology in vivo (i.e., complementation of yeast *tpt1*∆ by bacterial Tpt1s) underscore the value of our CthTpt1 structure as an initial template for the design of Tpt1 inhibitors as candidate antifungal agents. Occlusion of the NAD^+^ binding pocket (e.g., by an analog of NAD^+^ or ADP-ribose) and/or occupancy of the pocket for the RNA junction nucleobase (e.g., by a planar aromatic ring system) are potential means of interdicting Tpt1 function. A transition-state analog that interacts with the invariant Arg-His-Arg-Arg tetrad would be an ideal inhibitor on which to predicate drug design.

Finally, our good fortune in crystallizing Tpt1 in complex with ADP-ribose-1″-phosphate is the first crack in a longstanding conundrum anent the widespread presence of Tpt1 enzymes in bacterial proteomes. Because, the model bacterium *E. coli* has no yeast-like RNA ligase that would be expected to generate an internal 2′-PO_4_ RNA splice junction, it was not at all clear whether *E. coli* even has an endogenous substrate for its Tpt1 enzyme (known as KptA). Contributing to the riddle is the fact that genetic ablation of *E. coli* Tpt1/KptA does not affect bacterial growth^12^. To our knowledge, there has been no prior description of ADP-ribose-1″-phosphate as a metabolite in *E. coli* or any other bacterium. The presence of this molecule in the NAD^+^ site of our CthTpt1 crystals is most plausibly explained by its genesis during CthTpt1 expression in vivo, via CthTpt1-catalyzed ADP-ribosylation of an endogenous bacterial phospho-substrate, followed by phosphoryl transfer to yield ADP-ribose-1″,2″-cyclic phosphate that is hyrolyzed in situ to ADP-ribose-1″-phosphate. This provides evidence that a bacterium has an endogenous Tpt1 substrate. Two possibilities come to mind. First, that *E. coli* does have the capacity to form an RNA with an internal 2′-PO_4_ modification, via an as yet undefined enzyme, either by ligation of a 2′-PO_4_/3′-OH end to a 5′-PO_4_ end or by phosphorylation of an internal 2′-OH by a kinase. With respect to the ligation scenario, it is worth noting that *E. coli* has an RNA 2′,3′-cyclic phosphodiesterase ThpR that readily hydrolyzes a RNA>p end to an RNA 2′-monophosphate end^26^. Second, it is conceivable that *E. coli* has an endogenous non-RNA phospho-substrate upon which bacterial Tpt1 enzymes can act to form a dephosphorylated non-RNA product and ADP-ribose-1″,2″-cyclic phosphate. Whereas we cannot exclude the existence of a non-RNA phospho-substrate for bacterial Tpt1, we favor an endogenous RNA 2′-PO_4_ as the source of the 1″-PO_4_ in the crystal structure, in light of the observation that the RNA-like pAp moiety of CoA occupies a junction-binding site in the RNA lobe.

## Methods

### Recombinant Tpt1 proteins

His_6_-tagged CthTpt1 and CthTpt1-C46S mutant and His_6_-tagged RslTpt1 and RslTpt1-Ala mutants were produced in *E. coli* and purified from soluble bacterial extracts by sequential Ni-NTA agarose and gel-filtration chromatography steps^27^. Protein concentrations were determined by using the BioRad dye reagent with bovine serum albumin as the standard.

### Crystallization of CthTpt1 and structure determination

CthTpt1 and CthTpt1-C46S were concentrated by centrifugal ultrafiltration to 8.5 and 13 mg/ml, respectively, in 20 mM Tris-HCl, pH 8.0, 300 mM NaCl, 1 mM DTT, 5% glycerol. Crystals of CthTpt1 were grown by sitting drop vapor diffusion at 22 °C after mixing 1 µl of Tpt1 protein solution with 1 µl of precipitant solution containing 0.1 M MES, pH 6.0, 10% 2-methyl-2,4-pentanediol. Crystals appeared overnight and were cryoprotected with precipitant solution containing 25% glycerol before being flash frozen in liquid nitrogen. Crystals of CthTpt1-C46S were grown by sitting drop vapor diffusion at 22 °C after mixing 1 µl of Tpt1 protein solution with 1 µl of precipitant solution containing 0.2 M ammonium phosphate, 0.1 M Tris-HCl, pH 8.5, 50% 2-methyl-2,4-pentanediol. Crystals appeared after 5 days and were cryoprotected with precipitant solution containing 25% glycerol before being flash frozen in liquid nitrogen. Diffraction data to 1.4 Å and 1.55 Å resolution, respectively, were collected from single crystals of CthTpt1 (at a wavelength of 1.20510 Å) and CthTpt1-C46A (at a wavelength of 0.9791 Å) at APS beamline 24ID-C equipped with a Pilatus 6 M detector. The diffraction data were integrated in HKL2000^28^. The proteins crystallized in space group *P*6_1_22 with one CthTpt1 protomer in the asymmetric unit. Initial phases for CthTpt1 were obtained by molecular replacement in Phaser MR^29^ using the ApeTpt1 apoenzyme (pdb 1WFX) as the search model. Interactive model building was performed in O^30^. Refinement was accomplished in PHENIX^31^. Data collection and refinement statistics for CthTpt1 and CthTpt1-C46S are presented in Supplementary Table 1. In brief, the CthTpt1 structure at 1.4 Å resolution was refined to *R*_work_ and *R*_free_ values of 0.1616 and 0.1857, respectively, with 98.9% of residues in the favored regions of the Ramachandran plot (0 outliers). The CthTpt1-C46S structure at 1.55 Å resolution was refined to *R*_work_ and *R*_free_ values of 0.1727 and 0.1876, respectively, with 98.9% of residues in the favored regions of the Ramachandran plot (0 outliers).

### Test of *TPT1* function in vivo by plasmid shuffle

The *S. cerevisiae tpt1∆* haploid strain YBS501 (*MAT****a*** *ura3–1 ade2–1 trp1–1 his3–11,15 leu2–3,11–2 can1–100 tpt1::LEU2* p360-TPT1*)*, in which the *TPT1* ORF was deleted and replaced by *LEU2*, is dependent for viability on the p360-TPT1 plasmid (*CEN URA3 SceTPT1)*^16^. YBS501 was transformed with p413-RslTPT1 plasmids (*CEN HIS3*) expressing RslTpt1 or RslTpt1-Ala proteins under the control of the constitutive yeast *TPI1* promoter^5^ and with the empty *CEN HIS3* vector as negative control. Transformants were selected at 30 °C on His^−^ agar medium. Three individual His^+^ colonies were patched to His^−^ agar medium and cells from each isolate were then streaked on agar medium containing 0.75 mg/ml 5-FOA (5-fluoroorotic acid) and incubated at 30 °C. *RslTPT1* allele *H99A-T101A* that did not allow formation of FOA-resistant colonies after 7 days was deemed lethal in vivo. FOA-resistant *tpt1*∆ p413-RslTPT1 and *tpt1*∆ p413-RslTPT1-Ala colonies were grown in YPD-Ad (yeast, peptone, 2% dextrose, 0.1 mg/ml adenine) liquid medium at 30 °C to mid-log phase (*A*_600_ 0.6), then diluted to attain *A*_600_ of 0.1, and aliquots (3 µl) of serial fivefold dilutions were spotted on YPD agar plates and incubated at 20, 25, 30, 34, and 37 °C.

### Reporting summary

Further information on experimental design is available in the Nature Research Reporting Summary linked to this article.

## Supplementary information

Supplementary Information

Reporting Summary

## Data Availability

The additional data support the findings of this study are available from the corresponding author upon request. The atomic cordinates of the Tpt1 structures have been deposited in the Protein Data Bank with accession codes 6E3A and 6EDE. A Reporting Summary for this article is available as a Supplementary Information file.
