# Supplementary material for: Structure of tRNA splicing enzyme Tpt1 illuminates the mechanism of RNA 2′-PO4 recognition and ADP-ribosylation
Source: Nat Commun. 2019 Jan 15;10:218. doi: 10.1038/s41467-018-08211-9 (PMC6333775; doi:10.1038/s41467-018-08211-9)
Supplement: Supplementary file 1 — Supplementary Information [file 41467_2018_8211_MOESM1_ESM.pdf]

Supplementary Information

Structure of tRNA splicing enzyme Tpt1 illuminates the mechanism of RNA 2'-PO_4 recognition and ADP-ribosylation

Banerjee et al.

Supplementary Table 1 Data collection and refinement statistics

	Tpt1 (PDB 6E3A)	Tpt1-C46S (PDB 6EDE)
**Data collection**		
Space group	$P6_122$	$P6_122$
Cell dimensions		
a, *b*, *c* (Å)	53.37, 53.37, 308.9	53.28, 53.28, 308.39
α, β, γ (°)	90.00, 90.00, 120.00	90.00, 90.00. 120.00
Resolution (Å)	50.00-1.40 (1.42-1.40)*	50.00-1.55 (1.59-1.55)
R_{pim}	0.016 (0.252)	0.014 (0.208)
$I / \sigma I$	58.1 (2.1)	56.6 (2.8)
Completeness (%)	99.8 (97.8)	99.3 (85.8)
Redundancy	17.5 (10.9)	17.7 (9.8)
$CC_{1/2}$	0.998 (0.823)	0.99 (0.884)
**Refinement**		
Resolution (Å)	50.0-1.40	50.00-1.55
No. reflections	52908	38941
R_{work} / R_{free}	0.1616 / 0.1857	0.1727 / 0.1876
No. atoms		
Protein	1476	1459
Ligand/ion	88	40
Water	192	201
B-factors		
Protein	31.9	27.0
Ligand/ion	44.8	19.4
Water	42.9	40.4
R.m.s. deviations		
Bond lengths (Å)	0.015	0.005
Bond angles (°)	1.467	0.853

*Values in parentheses are for highest-resolution shell.

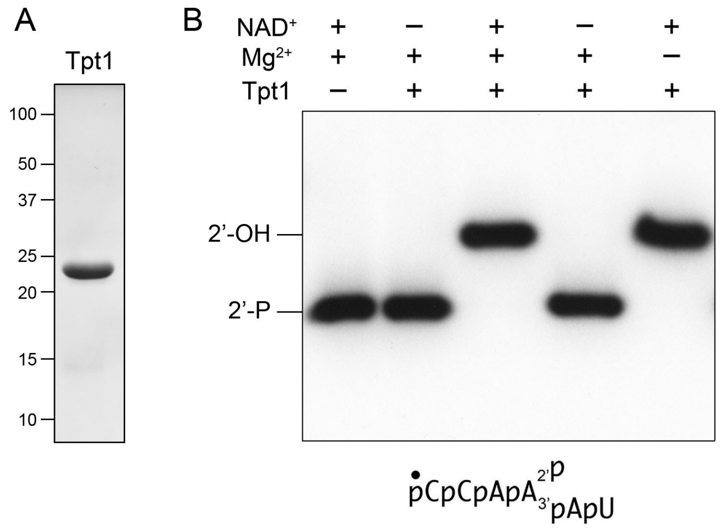

Supplementary Figure 1. RNA 2'-phosphotransferase activity of recombinant CthTpt1. (A) An aliquot (5 μg) of the CthTpt1 preparation was analyzed by SDS-PAGE. The Coomassie blue-stained gel is shown. The positions and sizes (kDa) of marker polypeptide are indicated at left. (B) Reaction mixtures (10 μl) containing 100 mM Tris-HCl, pH 7.5, 0.2 μM (2 pmol) 5' ^{32}P-labeled 6-mer 2'-PO_4 RNA (shown at bottom), 1 mM NAD^+ (where indicated by +), 5 mM $MgCl_2$ (where indicated by +), and 0.1 μM (1 pmol) CthTpt1 (where indicated by +), were incubated at 37˚C for 30 min. The reactions were quenched by adding three volumes of cold 90% formamide, 50 mM EDTA. The products were analyzed by urea-PAGE and visualized by autoradiography. The positions of the 6-mer 2'-PO_4 RNA substrate (2'-P) and 2'-OH RNA product (2'-OH) of the canonical Tpt1 reaction are indicated on the left.

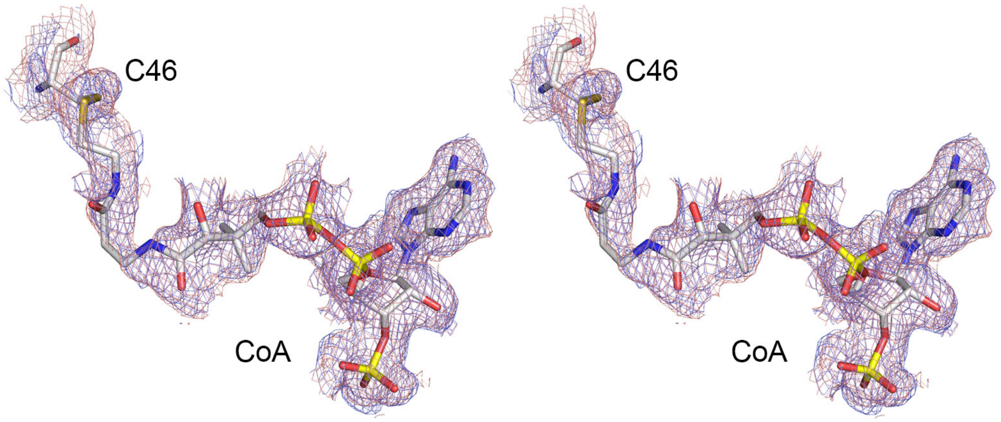

Supplementary Figure 2. Stereo view of SA omit maps of the coenzyme A (CoA) ligand. The Fo-Fc map, contoured at 0.9σ, is colored blue. The 2Fo-Fc map, contoured at 0.35σ, is colored red. The maps were calculated with CoA and ADPR-1"-PO_4 omitted from the model. The 2Fo-Fc map shows that CoA and Cys46 (shown as stick models) are linked via a disulfide bond.

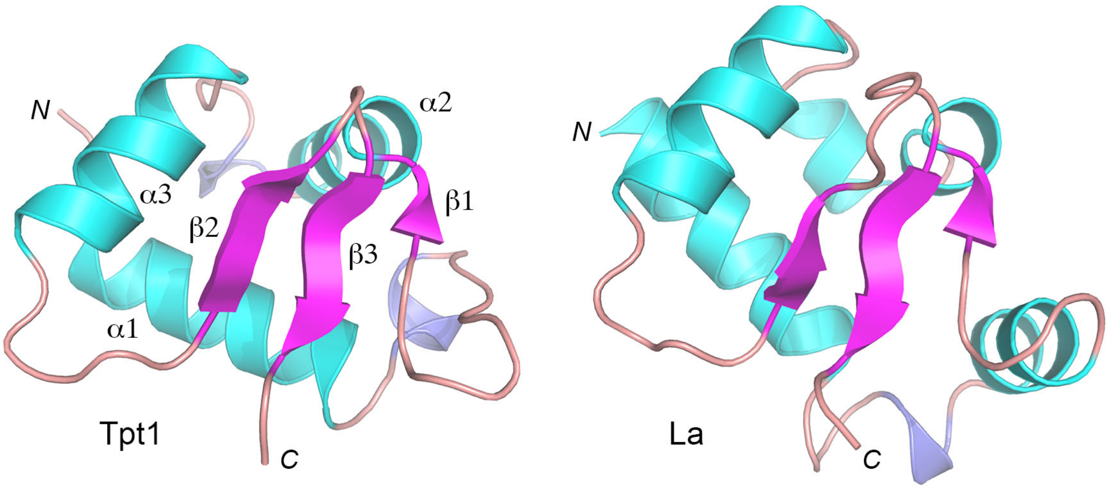

Supplementary Figure 3. The Tpt1 RNA lobe is a winged helix fold with similarity to human La protein. Side-by-side alignment of the winged helix modules of CthTpt1 (with secondary structure elements labeled) and human La protein (pdb 1YTY).

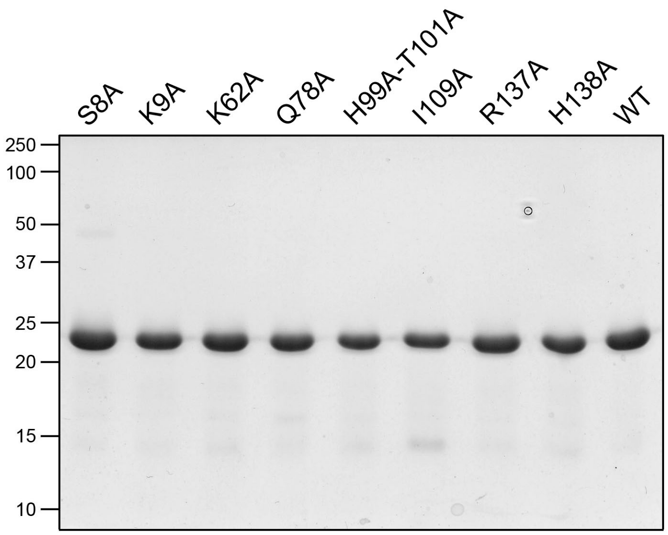

Supplementary Figure 4. Tpt1-Ala mutants. Aliquots (~5 μg) of purified recombinant wild-type RslTpt1 and the indicated Ala-mutants were analyzed by SDS-PAGE. The Coomassie blue-stained gel is shown. The positions and sizes (kDa) of marker proteins are indicated on the left.
